# Magnification of digital hip radiographs differs between clinical workplaces

**DOI:** 10.1371/journal.pone.0188743

**Published:** 2017-11-30

**Authors:** Jana Hornová, Pavel Růžička, Maroš Hrubina, Eduard Šťastný, Andrea Košková, Petr Fulín, Jiří Gallo, Matej Daniel

**Affiliations:** 1 Laboratory of Biomechanics, Faculty of Mechanical Engineering, Czech Technical University in Prague, Prague, Czech Republic; 2 Department of Orthopaedics, Hospital Pelhřimov, Pelhřimov, Czech Republic; 3 Department of Paediatric and Adult Orthopaedic Surgery and Traumatology, Second Faculty of Medicine Charles University and Teaching Hospital Motol in Prague, Prague, Czech Republic; 4 Department of Radiology, Hospital Jablonec nad Nisou, Jablonec nad Nisou, Czech Republic; 5 Department of Orthopaedic, First Faculty of Medicine Charles University and Teaching Hospital Motol in Prague, Prague, Czech Republic; 6 Department of Orthopaedics, Faculty of Medicine and Dentistry, Palacký University Olomouc, Teaching Hospital Olomouc, Olomouc, Czech Republic; University of Groningen, University Medical Center Groningen, NETHERLANDS

## Abstract

Preoperative planning for total hip arthroplasty includes templating on anteroposterior radiographs. It is necessary to consider radiographic magnification in order to scale templates accurately. Studies dealing with hip templating report different values of radiographic magnification. It is not clear if the observed difference in magnification between the studies is caused by variability in studied groups, methodology or instrumentation. We hypothesize that there is a difference in magnification between clinical workplaces. Within this study, radiographic magnification was estimated on 337 radiographs of patients after total hip surgery from five orthopaedic departments in the Czech Republic. Magnification was determined for each patient as a ratio between diameter of implanted femoral head measured on radiogram and its true size. One-way ANOVA revealed significant differences in magnification between workplaces (F(4,332) = 132, p≤0.001). These results suggest that radiographic magnification depends on the workplace where it is taken or more precisely on radiographic device. It indicates potential limits in generalizability of results of studies dealing with preoperative planning accuracy to other institutions.

## Introduction

It is recommended practice in total hip arthroplasty (THA) preoperative planning to template the hip radiographs using either acetate templates or digital templating software [1]. The main goal of hip templating is to estimate the size, position and insertion depth of both acetabular and femoral components that will reproduce hip biomechanics [2]. Accurate templating can reduce complications such as instability, leg length discrepancy, periprosthetic fracture, prosthetic loosening, and loss of bone stock [3].

The templating procedure has potential inherent flaws resulting from unknown radiographic magnification [4]. Preoperative radiographs could be performed with a magnification marker that allows scaling of the radiograph [5]. In recent studies, Archibeck et al., 2016 [4] and Franken et al., 2010 [6] showed that taking fixed 120% and 121% magnification, respectively could be more accurate than use of magnification marker. The value of magnification around 120% has also been observed in the clinical total hip arthroplasty studies (Table 1) where the known diameter of implanted femoral head is used as a magnification marker. However, there is obvious scattering in the observed hip magnification between and within the studies in Table 1. It is not clear whether differences between the studies are caused by variations between studied groups, instrumentation or adopted methodology or are just a result of random sample selection from the same population.

**Table 1:** Radiographic magnification determined in clinical studies. Femoral head after THA is used as a magnification reference.

			Radiographic magnification	
Reference	Num. of patients		Mean	Range
Archibeck et al. (2016) [4]		100	121%	(117%– 127%)
Bayne et al. (2009) [12]		106	120%	–
Boese et al. (2015) [7]		100	122.5%	(106%– 130%)
Descamps et al. (2010) [1]		100	126.1%	(121%– 130%)
Heinert et al. (2009) [13]		22	120.0%	(109%– 128%)
King et al. (2009) [14]		50	118.3%	(113%– 133%)

**Fig 1:**
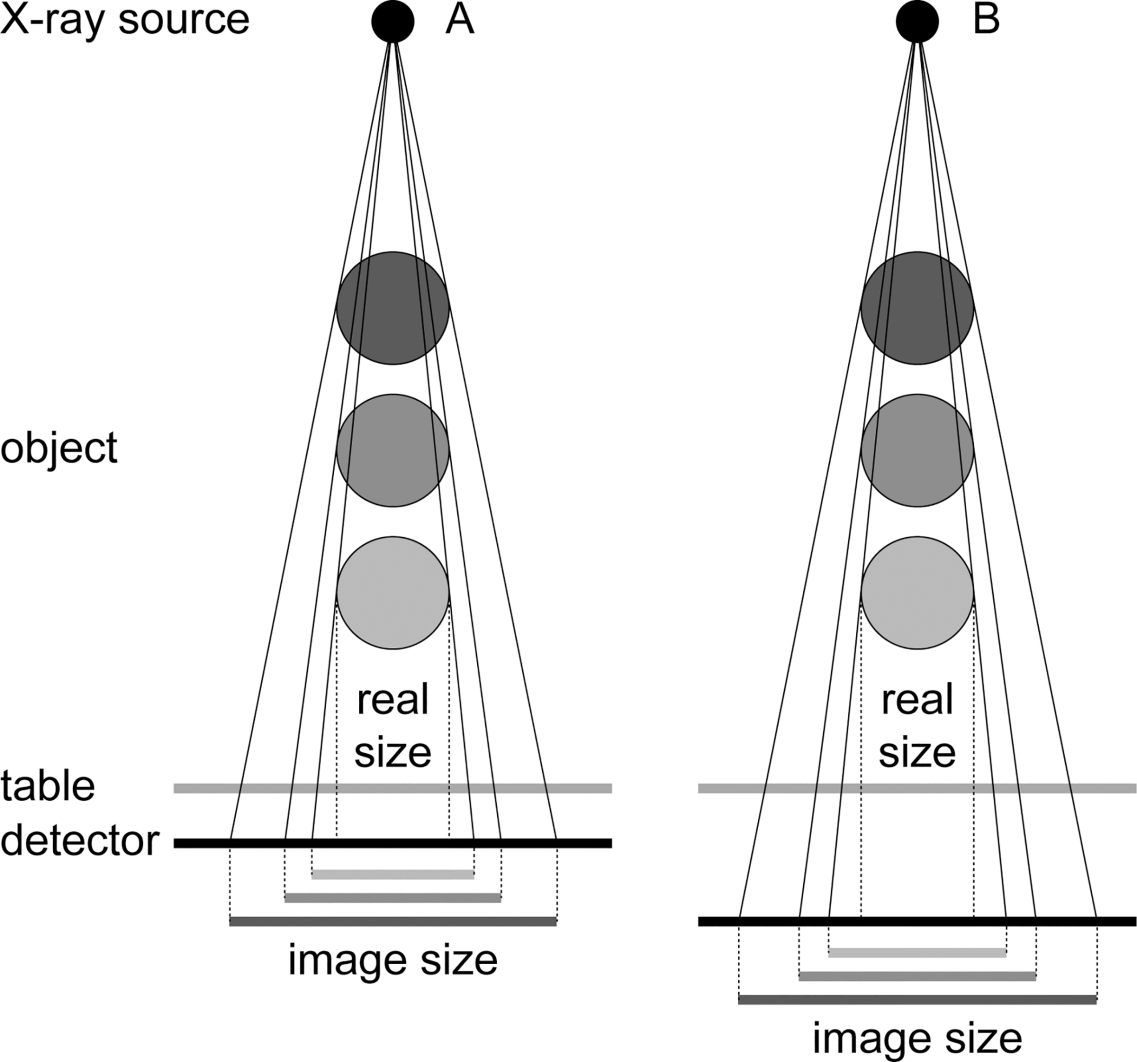
Schematic diagram showing radiological magnification. (A and B) various positions of hip joint over the table, (A vs. B) various radiological setups.

The accuracy of templating has been evaluated with respect to the marker type and position [4, 7, 8], template type (acetate vs. digital) [3], sex, age, body mass index and Harris Hip Score [9–11]. However, there is limited information whether results from one workplace dealing with certain radiological setup could be quantitatively transferred to another workplace. As radiographic magnification depends on radiological setup (Fig 1), we hypothesize that the magnification of radiographs depends on the workplace where it is taken. To test this hypothesis, we evaluated magnification of radiographs of patients after THA obtained from several hospitals but within the same population.

## Patients and methods

The study cohort in the Czech Republic was chosen for the homogeneity of population, with over 97% Caucasians and low migration. 372 AP radiographs obtained during regular follow-ups after total hip replacement were studied. The data were gathered from November 2012 to May 2016 at five orthopaedic departments in the Czech Republic, denoted as A-E (Table 2). A single radiographic device specified in Table 2 was used at each department. All AP pelvic radiographs were obtained with the patient in a supine position. Radiographs with incomplete records, e.g. missing femoral head radius and poor quality of radiographs (motion artifacts, not clearly visible femoral head) were excluded from the study (35 images). The study included 337 images (Table 2).

**Table 2:** Clinics, X-ray devices and patients included in the study.

	Institution	X-ray machine	Total	Male	Female
A	Department of Radiology, Hospital Jablonec nad Nisou	Philips DigitalDiagnost	52	22	30
B	Department of Orthopaedics, Second Faculty of Medicine, Charles University in Prague and Teaching Hospital Motol	Kodak DirectView DR 7500, Fujifilm scanner	59	23	36
C	Department of Orthopaedics, Hospital in Pelhřimov	Canon CXDI	108	44	64
D	Department of Orthopaedics, First Faculty of Medicine, Charles University in Prague and Teaching Hospital Motol	Philips Bucky Diagnost	43	20	23
E	Department of Orthopaedics, Faculty of Medicine and Dentistry, Teaching Hospital, Palacký University Olomouc	Agfa CR 85 scanner	75	24	51

The magnification of the radiograph was calculated by using the femoral head of known size as reference. The analysis was performed using ImageJ [15], where first, the actual pixel size in millimeters was obtained either from DICOM header or from scale at the radiograph and second, the diameter of femoral head was measured as a diameter of circle conforming to the contour of the femoral head. The magnification was computed as ratio between measured and real femoral head diameter. For detailed method of magnification estimation from DICOM images see Supplementary file (S1 Text). The measurements were performed by single observer.

To assess the validity of the method for radiographic magnification estimation, five independent, untrained and blinded observers analyzed set of 50 randomly selected radiographs using a computer-based technique (S1 Text, inter-class variance). Repeated measures were performed 8 months after the first analysis by one observer on the same set of 50 radiographs, blinded to the previous results (intra-class variance).

To test a repeatability of radiographic measurement setup, digital radiographs in workplaces A and B were taken with magnification marker. Magnification marker is 20-mm metallic sphere placed on the stand at fixed height 60 mm above the table. The magnification of marker was measured by the same method as the magnification of femoral head.

Statistical analysis was performed statistical software R (R Foundation for Statistical Computing, Vienna, Austria). The Shapiro-Wilk test was used to determine whether the data was normally distributed. One-way analysis of variance (ANOVA) was used for normally distributed continuous variables and Kruskal-Wallis test was used for non-normally distributed continuous variables. The results were then further analysed with Tukey post-hoc tests. Inter- and intra-observer variability were measured using the intra-class correlation coefficient ICC (model 2,1 as described by Shrout and Fleiss [16]). The value of intra-class correlation coefficient from 0.00 to 0.20 was considered slight, 0.21 to 0.40 was considered fair, 0.41 to 0.60 was considered moderate, 0.61 to 0.80 was considered substantial and 0.81 to 1.00 was considered excellent [17]. The level of significance was set at p < 0.01. Data of radiographic magnification are presented as mean averages, range and standard deviations.

## Results

There was an excellent agreement between observers and between measurements on estimated radiographic magnification (inter-rater ICC = 0.949; 95% confidence interval 0.924–0.968 and intra-rater ICC = 0.988; 95% confidence interval 0.979–0.993). The magnification of external marker measured at clinic A (111.5% ± 0.5%) and B (109.9% ± 0.5%) exhibits almost constant value with low variation between radiographs measured at the same clinic. It confirms identical radiological setup used for all patients at given workplace. The height and weight of patients does not differ between the workplaces (Kruskal-Wallis test p = 0.304 and p = 0.991, respectively).

**Fig 2:**
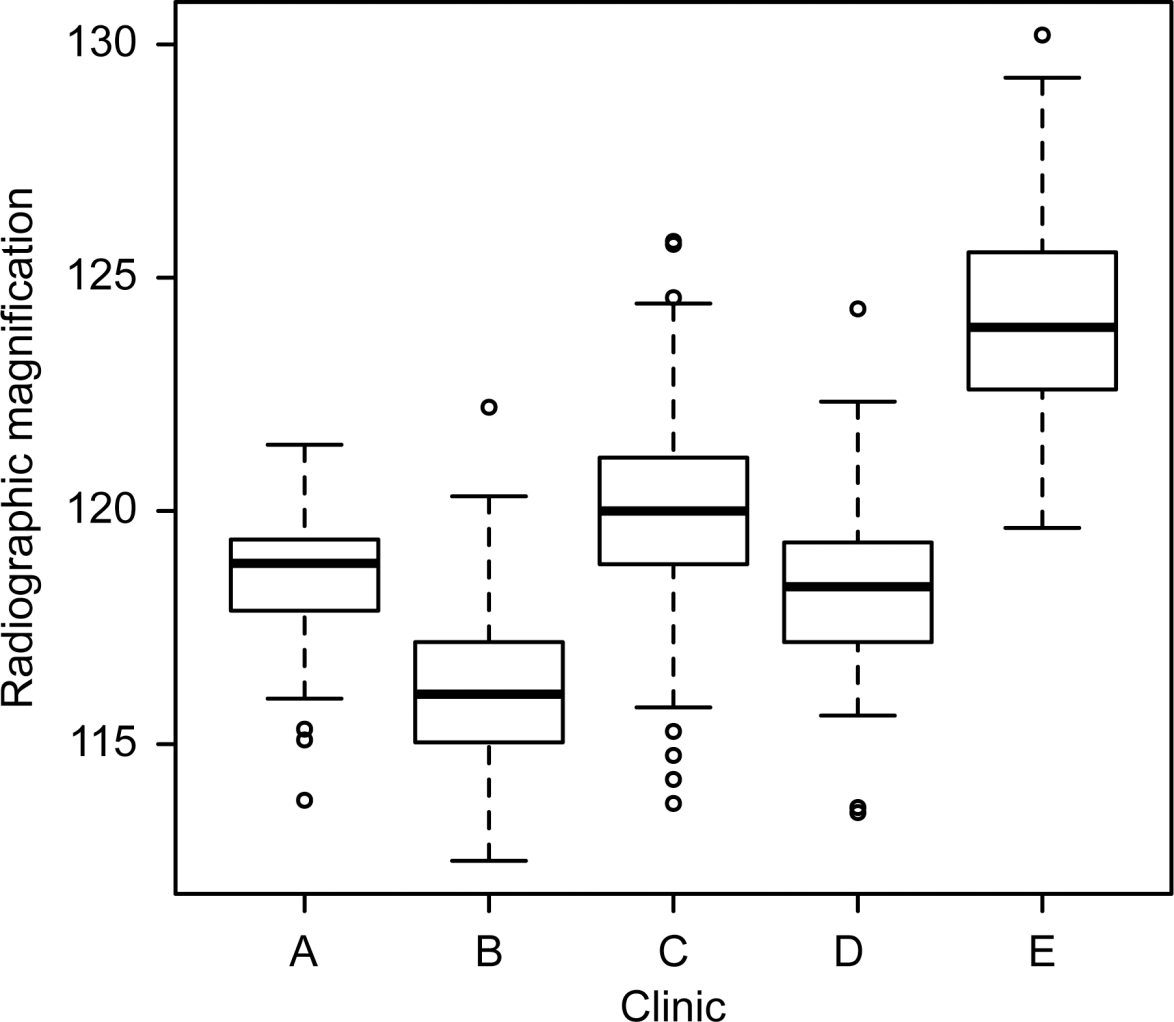
Boxplot of radiographic magnification. Hip radiographic magnification measured from radiographs of patients after total hip replacement at five hospitals.

Table 3 shows radiographic magnifications obtained at five different workplaces. There is a significant effect of the choice of clinic on radiographic magnification (**ANOVA** F(4,332) = 132, p≤0.001), Fig 2. Post-hoc comparisons indicates significantly different radiographic magnification between all hospitals (p<0.002) except for difference in magnification between clinics A and D (p = 0.99).

**Table 3:** Radiographic magnification obtained at five workplaces.

Clinic	Mean	±	std	Range
A	118.6%	±	1.7%	(113.8%– 121.4%)
B	116.2%	±	1.8%	(112.5%– 122.2%)
C	119.9%	±	2.4%	(113.7%– 125.8%)
D	118.5%	±	2.1%	(113.5%– 124.3%)
E	124.2%	±	2.2%	(119.6%– 130.2%)

## Discussion

A crucial step in total hip templating is scaling the template to the magnification of radiograph. The templates are mostly provided prescaled to 20% [4], but the actual magnification is known to vary among patients. The magnification of hip radiograph depends on mutual position of X-ray source, patient and detector plane [13] that might depends on construction of particular X-ray device. This study was intended to estimate, if there is a significant variation of magnification between clinical workplaces.

An external marker at a fixed height over the table was used to ensure repeatability of measurements. Almost constant magnification of the external marker shows that the position of X-ray source, table and detector is identical for all patients [1] as is also required by radiological standards [18]. Lower magnification of external marker observed in present study is a result of placing the marker below the coronal plane of the hip as was also observed by Archibeck et al., 2016 [4]. Certain variation in external marker magnification could be caused by different lateral shift [5].

The magnification of radiographs observed in this study is within range obtained in previous studies (Table 1). Based on our results (Fig 2), we may conclude that differences between studies listed in Table 1 could also be caused by a difference in radiological magnification among clinical workplaces conditioned primarily by radiographic device construction. It is reasonable to expect that the internal construction of device, e.g. distance between the detector and the table, will affect the magnification as show in Fig 1. The range of observed magnification shows that there are other effects that may influence the radiographic magnification, e.g. distance between the femoral head and the table (Fig 1). These effects could be related to individual body habitus that causes vertical shift [7] or lateral shift of the hip in the projected beam [5].

The implanted femoral head of THA is considered to be the most accurate method for magnification estimation from hip radiographs. Nevertheless, internal markers are not available during THA planning and therefore external markers must be adopted. It is recommended to place marker at the level of hip coronal plane which could only be roughly estimated from greater trochanter position. As the marker must also be placed out of the body, lateral shift causes magnification distortion. These imperfections in marker placements introduce 6% error in radiographic magnification with range from -5% to 15% as shown by Archibeck et al., 2016 [4]. Also Leung et al., 2015 [19] and Franken et al., 2010 [6] report error estimated using magnification markers to be 7% and 2.5%, respectively. The studies of Archibeck et al., 2016 [4], Leung et al., 2015 [19] and Franken et al., 2010 [6] also show that taking a fixed value of magnification will reduce magnification error to less than 2% in all studies.

Archibeck et al., 2016 [4] proposed taking fixed 120% magnification instead of use of magnification marker in preoperative templating. Our results indicate that instead of taking fixed value of 120% for all workplaces, it would be more accurate to determine baseline magnification at each workplace separately. Data from archives of follow-ups could be used as proposed in the presented study. However, it should be noted, that when processing radiographs from various sources as in the presented study, it is important to pay attention to data consistency. For example, DICOM image header contains two references to pixel size: Imager Pixel Spacing Tag and Pixel Spacing Tag. Imager Pixel Spacing Tag provides information on physical distance measured at the front plane of the image receptor, while the Pixel Spacing Tag is filled automatically by the device making assumptions on magnification without documenting the nature of the correction. The correction of magnification set by device (e.g. 5% at clinic D) is not accurate with respect to the magnification of femoral head. While the correct information is given by the Imaginer Pixel Spacing, the Pixel Spacing value is used mostly to present a reference scale on DICOM images and on computer screen that might be misleading in templating presented by Petretta et al., 2015 [3].

The clinically tolerable margin of error depends on the steps between implant sizes. Franken et al., 2010 [6] showed that an exact template for a specific implant size would require magnification error limits below 2% for ABG-II cementless standard components. Our results show that maximum difference in mean magnification between workplaces can be as high as 8% (Table 3).

## Conclusions

Our study supplements previous studies by showing that magnification of hip radiographs depends not only on marker and patient-specific factors [7, 9, 10], but also on clinical workplace or more precisely on radiographic device. It indicates potential limits in generalizability of results of studies dealing with preoperative planning accuracy to other institutions. We may conclude that quantitative results on hip radiographic magnification for templating, such as optimal value of fixed magnification, cannot be simply transferred from one workplace to another.

## Supporting information

S1 Dataset Measurements of hip magnification. (TXT)

S2 Dataset Inter-personal variability study. (TXT)

S3 Dataset Intra-personal variability study. (TXT)

S1 Text THA radiographic magnification assessment using ImageJ software. Step-by-step guide.
(PDF)
