# Supplementary material for: Magnification of digital hip radiographs differs between clinical workplaces
Source: PLoS One. 2017 Nov 30;12(11):e0188743. doi: 10.1371/journal.pone.0188743 (PMC5708766; doi:10.1371/journal.pone.0188743)
Supplement: S1 Text — Step-by-step guide. (PDF) [file pone.0188743.s004.pdf]

Supporting Information

# THA radiographic magnification assessment

# using ImageJ software.

# Step-by-step guide.

This document describes the step-by-step workflow for estimating the radiographic magnification from .dicom images of patients after total hip arthroplasty. It is assumed that the real size of implanted femoral head is known. The ImageJ software, available at https://imagej.net/Downloads is used for measurements.

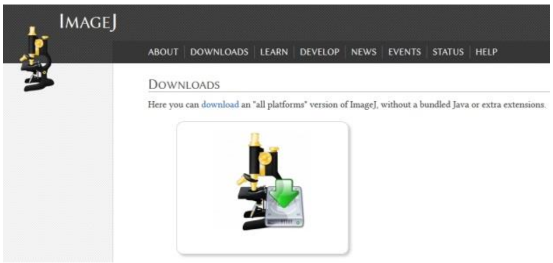


When the downloaded .zip file is extracted, the ImageJ can be run just by start an appropriate .exe file. No installation is necessary.

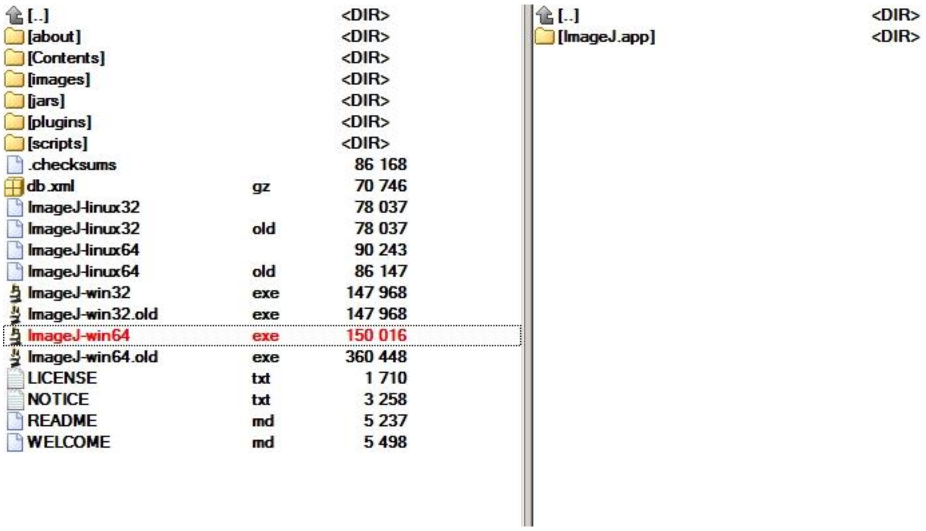


This is how ImageJ looks.

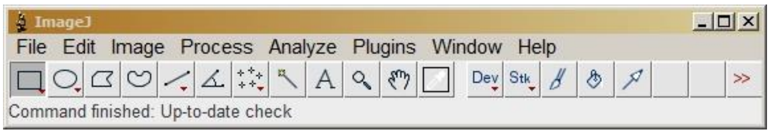

Use File -> Open for opening an image .dicom file. When this procedure does not work, File -> Import -> Image could help.

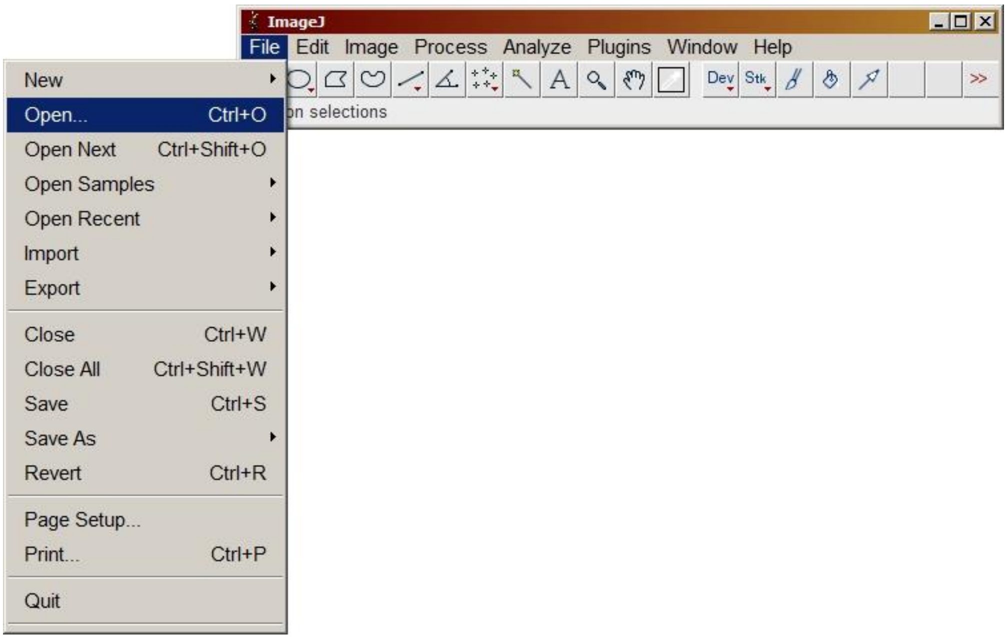


The image window is opened subsequently. The main image properties are displayed at the top of the window.

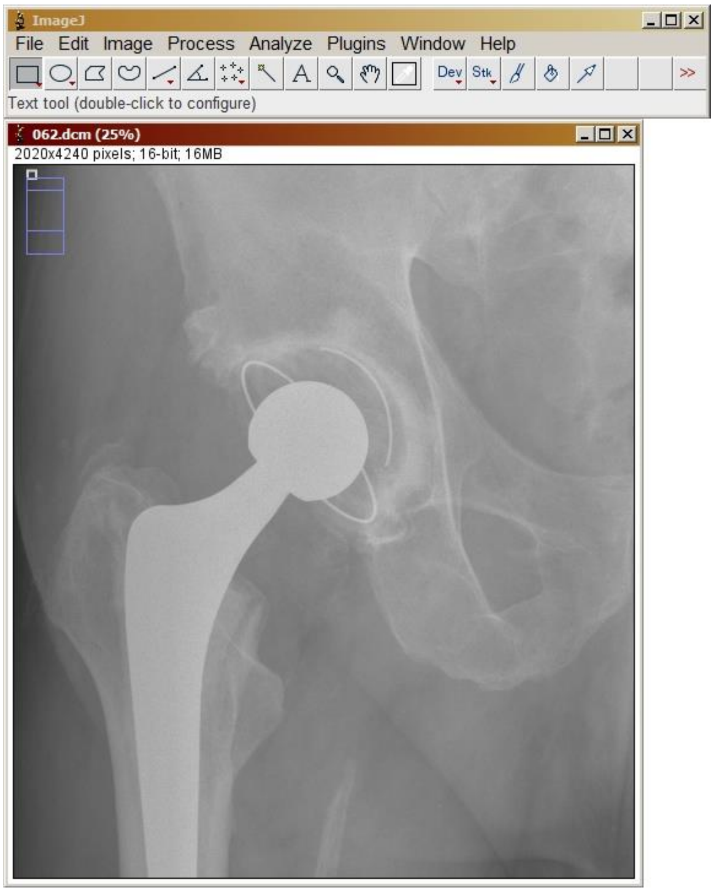

It’s possible to obtain the complete image information using: Image -> Show Info this will display tags with unique numbers, the name of each property and the appropriate value.

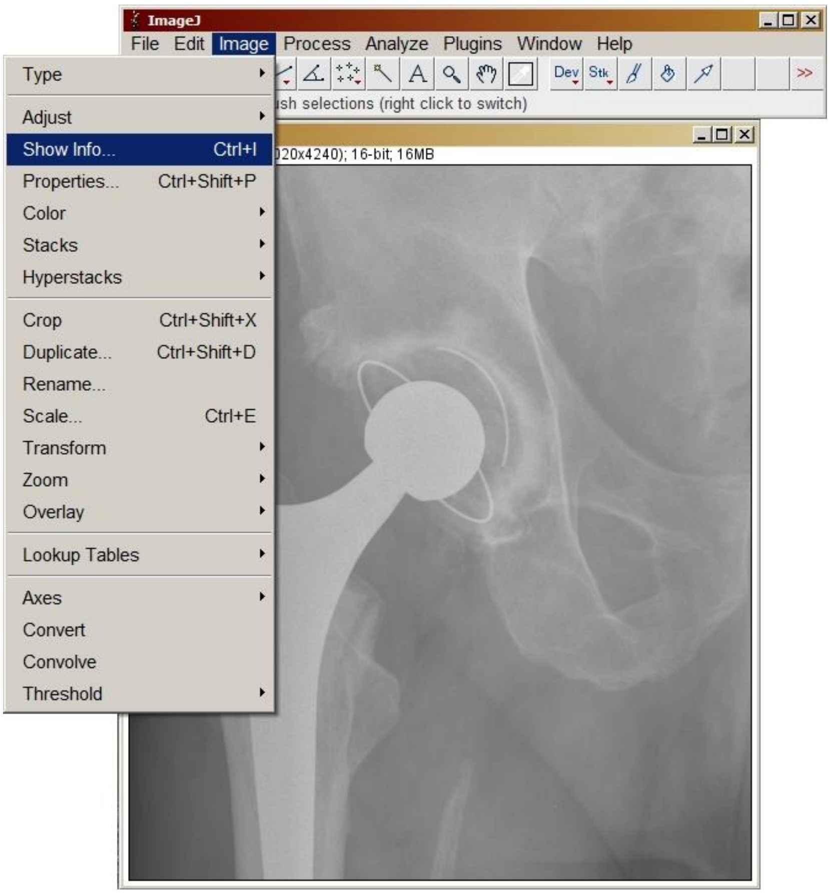


The pixel size is essential information, numbered (0018,1164 Imager Pixel Spacing).

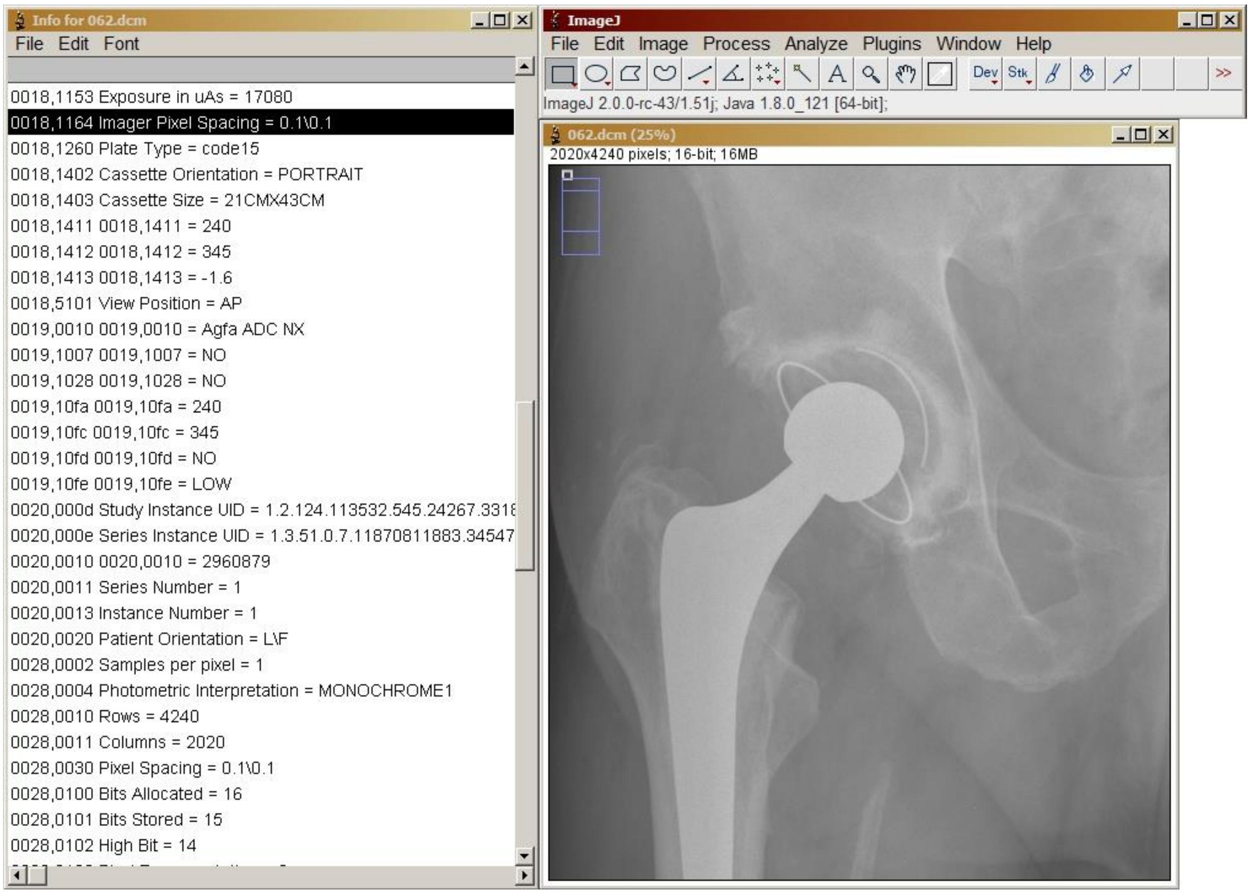

Do not confuse the tag (0018,1164) with tag number (0028,0030 Pixel Spacing). As showed on the example above, this second tag could display pixel size that has already been scaled.

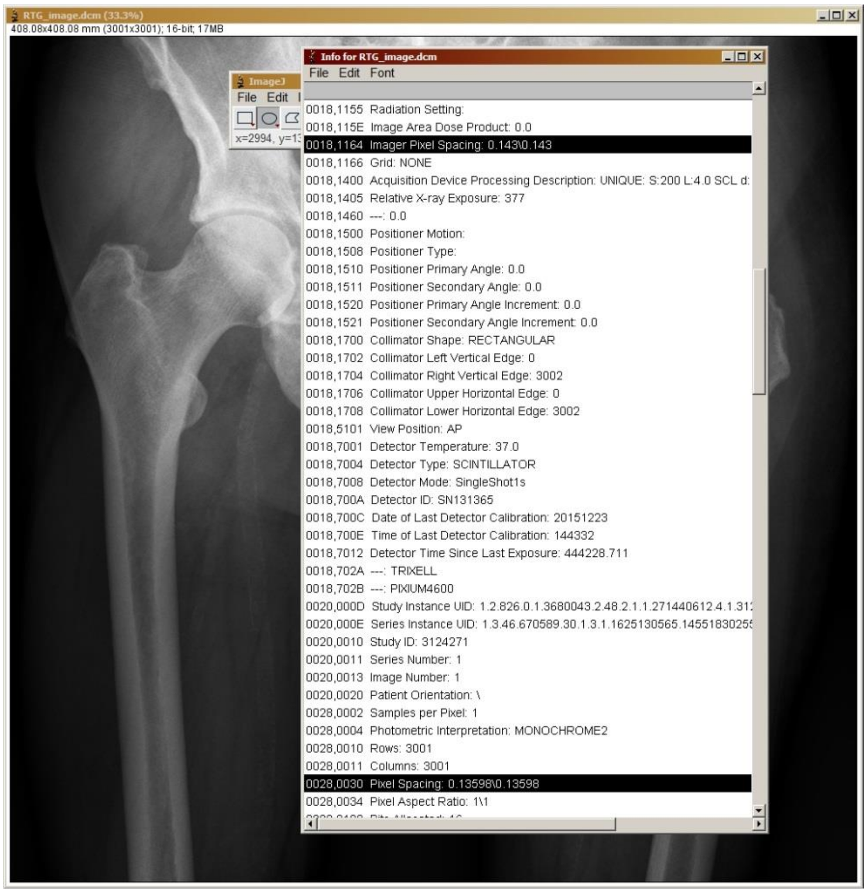


To ensure correct the dimensions during measurement, scale properties needs to be set at Analyze -> Set Scale...

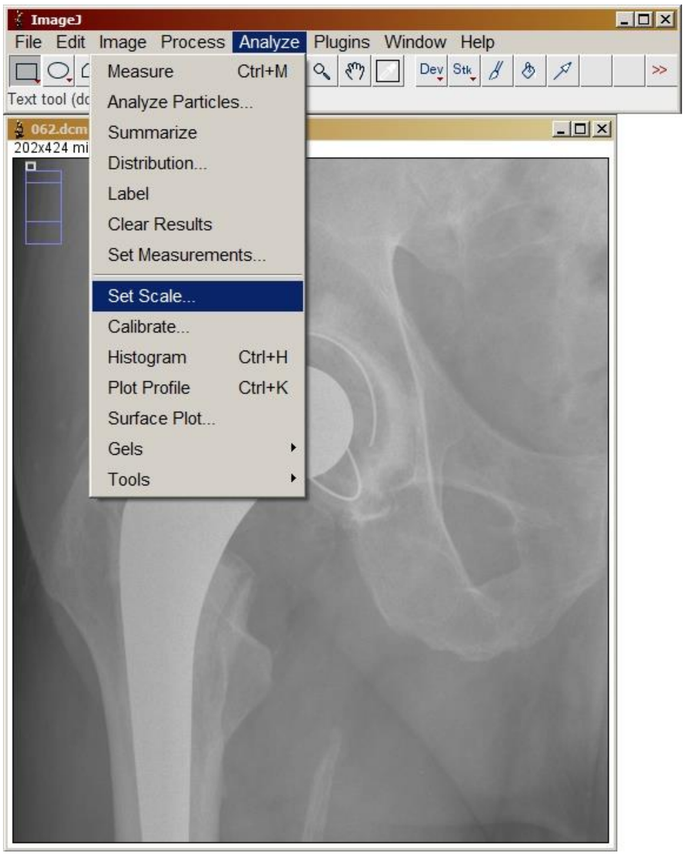

Set the true pixel size, value from tag (0018,1164 Imager Pixel Spacing). The unit has to be entered manually but it is shown completed for illustration purposes.

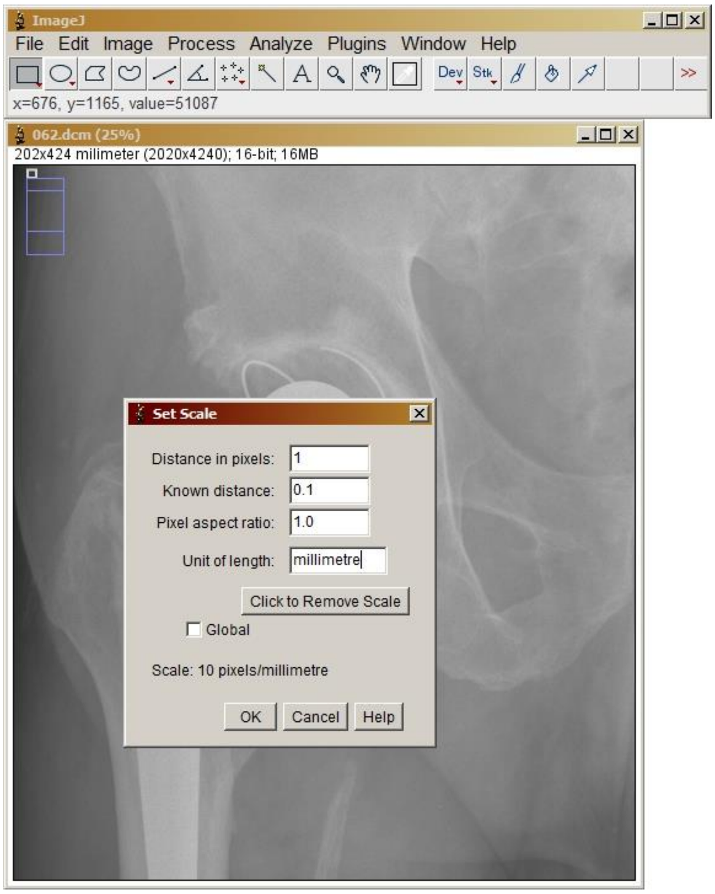


To set the required dimension it is possible to use: Analyze -> Set Measurements...

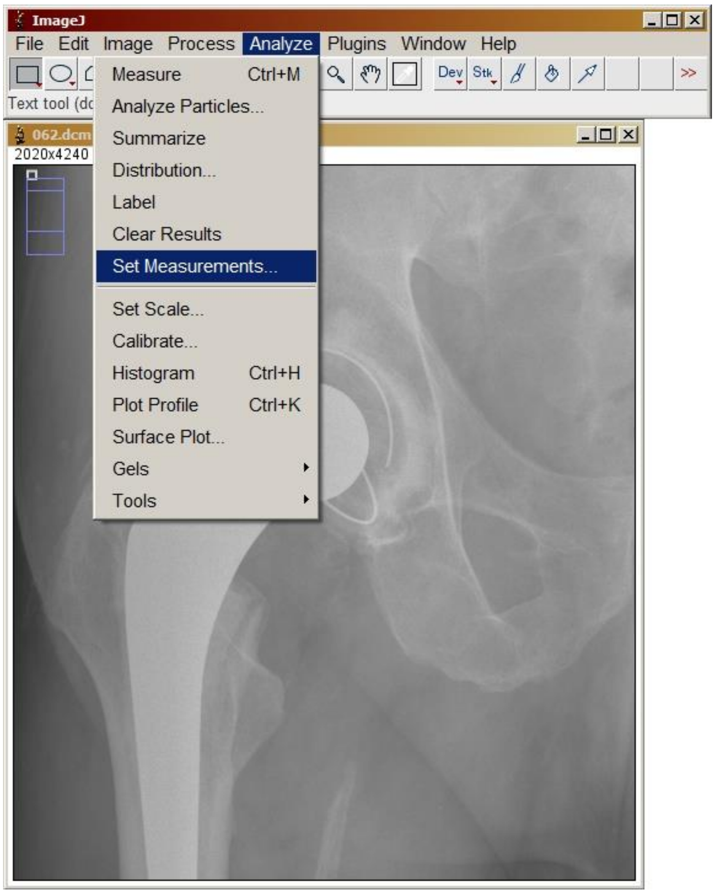

Choose Fit ellipse.

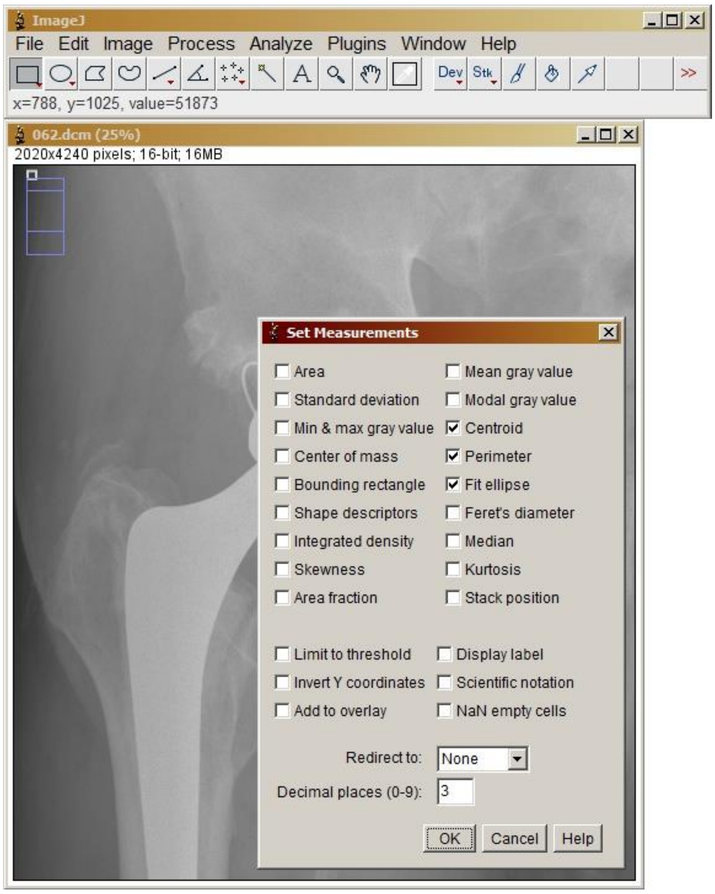


Zoom into the image for better visibility using: Image -> Zoom -> In/Out or just press + or -.

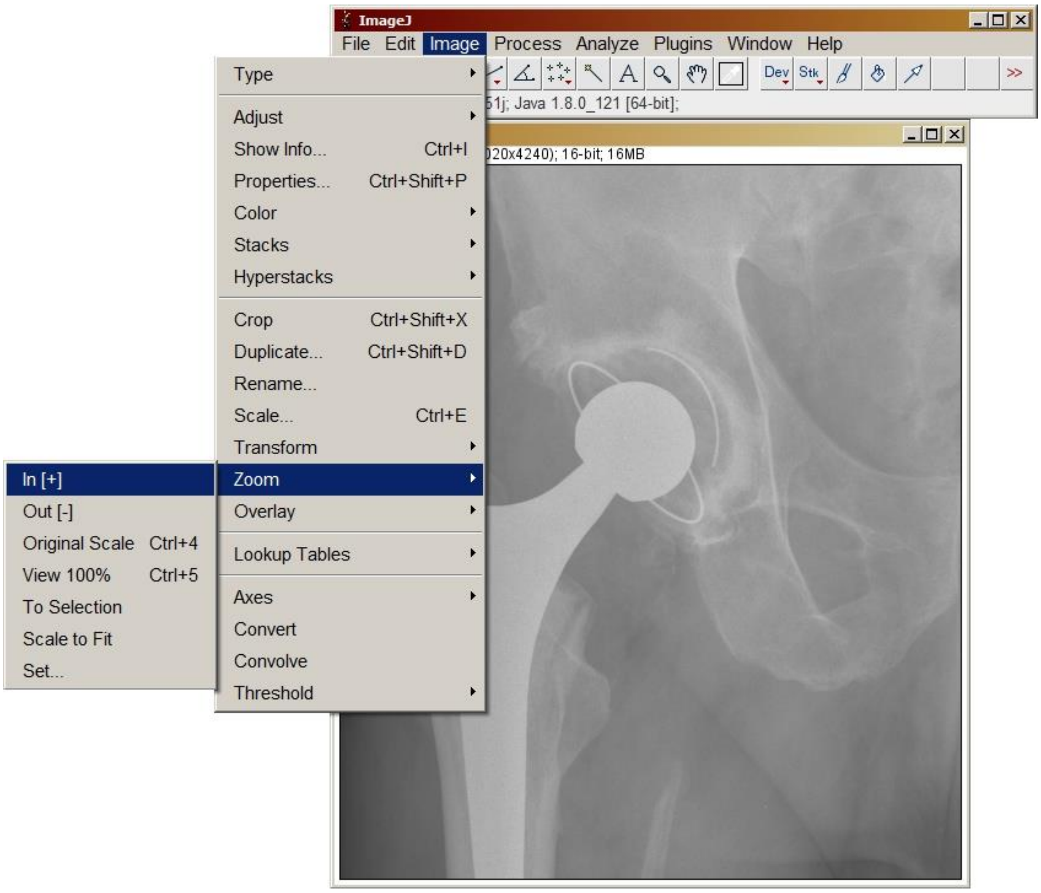

Select the Oval tool. The oval tool icon is on the second line, and the second pictogram from the left.

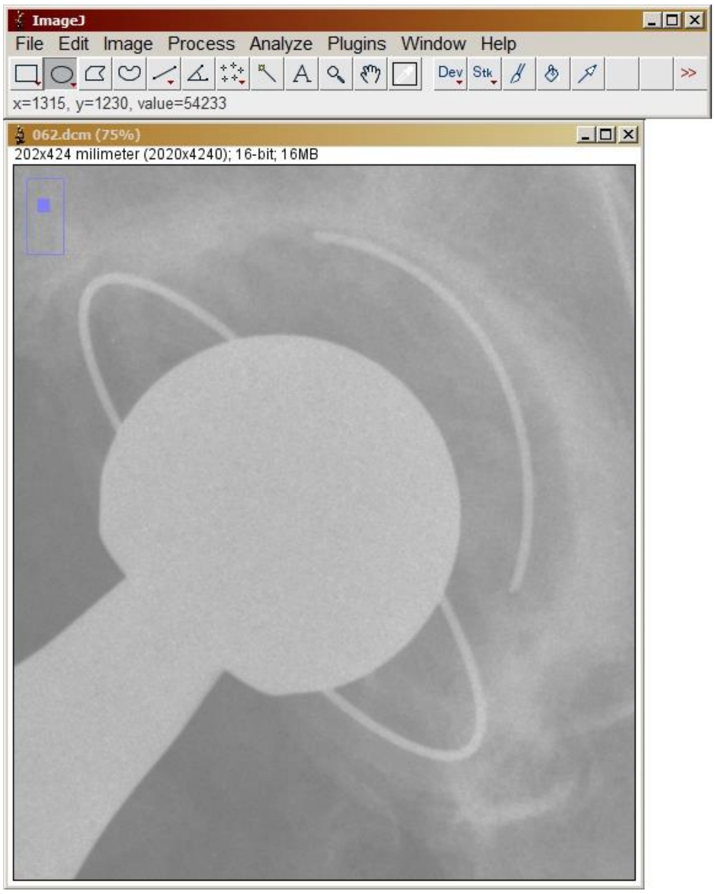


To draw a circle click, press Shift and drag, then second click. Without Shift an ellipse is obtained.

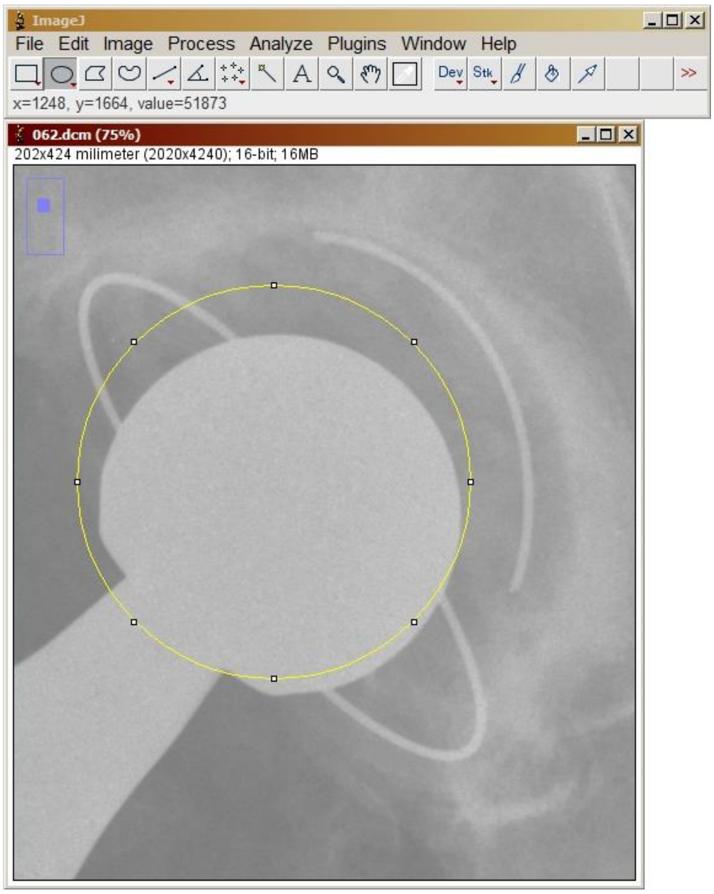

By dragging the small points together whilst holding Shift the circle can be adjusted to the femoral head contour.

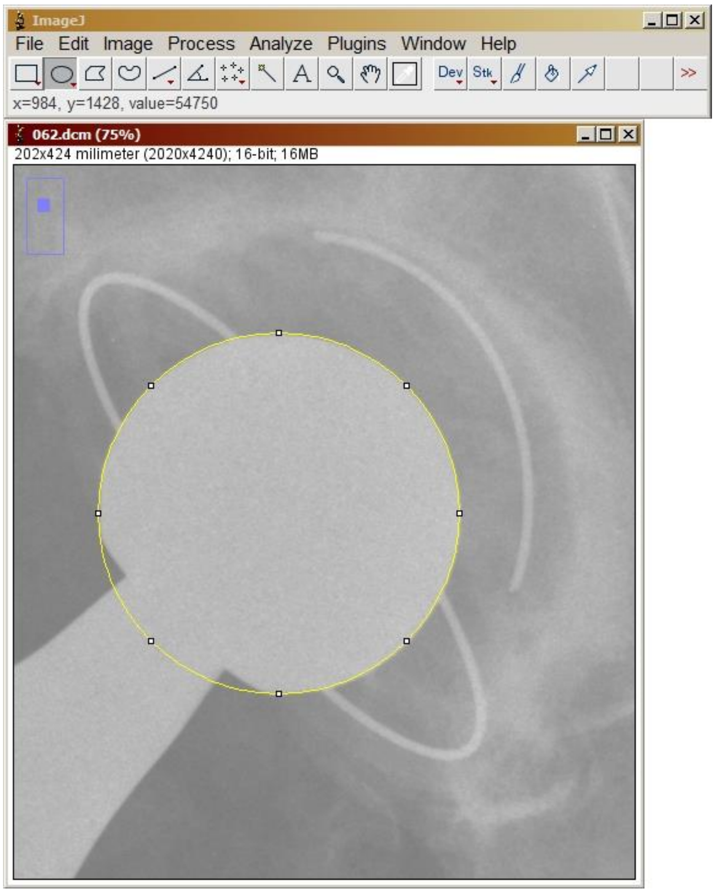


Open a result window displaying measurements by using: Analyze -> Measure.

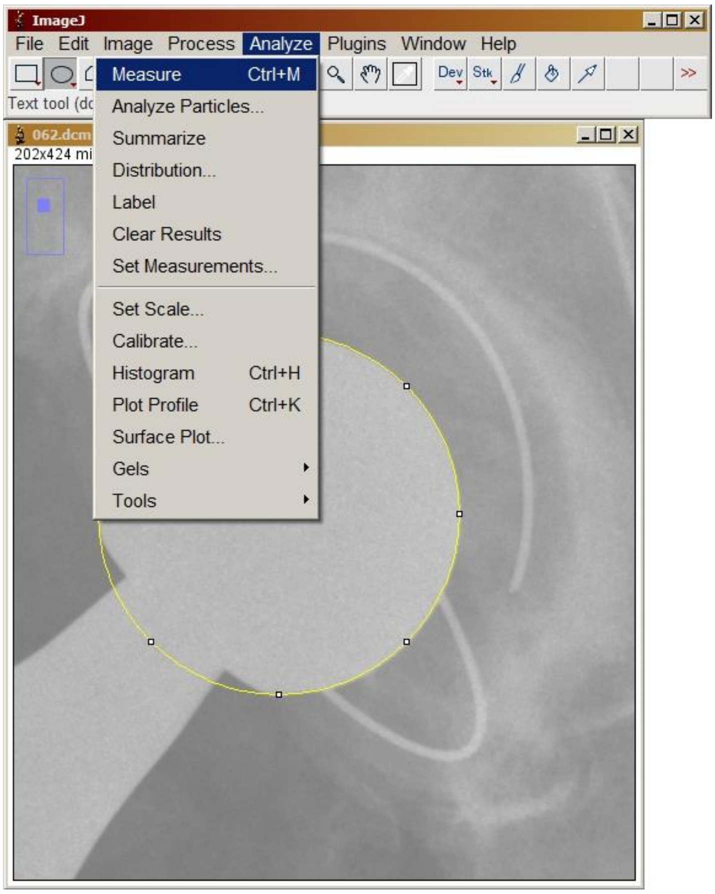

The value of the diameter is in the Major (or Minor) columns. These values will be the same if a circle is drawn and they will be different if ellipse is drawn.

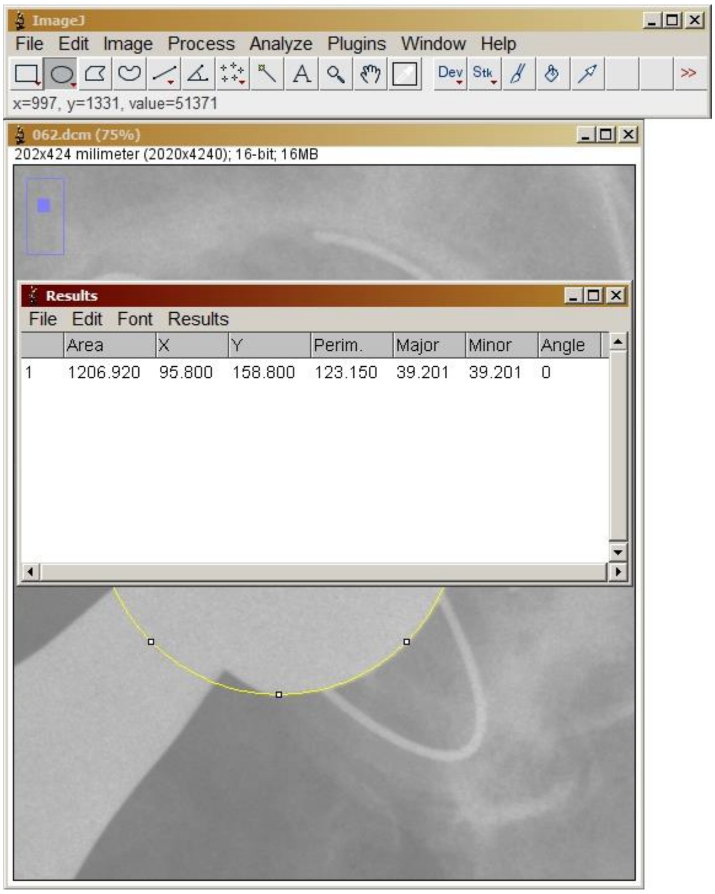


The Major dimension describes image femoral head size in millimeters. Magnification can be calculated as

$$\text{Magnification} = \frac{measured\ size}{real\ size} \cdot 100 = \frac{39.201}{32} \cdot 100 = 122.5\ \%.$$
